# High-resolution genome-wide analysis identified recurrent genetic alterations in NK/T-cell lymphoma, nasal type, which are associated with disease progression

**DOI:** 10.1007/s12032-014-0071-z

**Published:** 2014-06-22

**Authors:** Lin Sun, Min Li, Xin Huang, Jiaosheng Xu, Zifen Gao, Cuiling Liu

**Affiliations:** 1 Department of Pathology, School of Basic Medical Sciences, Peking University Health Science Center, Beijing, 100191 People’s Republic of China; 2 Department of Dermatology, Beijing Children’s Hospital, Beijing, 100045 People’s Republic of China

**Keywords:** Extranodal NK/T-cell lymphoma, nasal type, Array CGH, Genetic alterations, 8p11.23

## Abstract

Extranodal NK/T-cell lymphoma, nasal type, is an aggressive mature NK-cell/T-cell lymphoma. Using array-based comparative genomic hybridization (array CGH) assays, we screened genomic alterations and potential candidate genes implicated in pathogenesis, progression, and prognosis. Our array CGH analysis detected an average of 83 chromosomal aberrations in 13 cases, ranging from 0 to 387. There were 177 recurrent chromosomal gains and 35 recurrent losses. Eleven gains and 14 losses were detected in more than 30 % of the cases, including gains of 3q26.1, 7q34, and 8q24.3 and losses of 15q24.2, 19q13.32, 5p13.2, and 14q21.1. The most common losses were observed in the 15q24.2 and 19q13.32 regions (9 cases, 69.2 %, respectively). Loss of 8p11.23 was associated with significant poor survival (*P* = 0.024). Five out of six patients with the loss of 8p11.23 died within 8 months after initial diagnosis with a median survival of 6 months. Several candidate genes were identified in the regions with frequent chromosomal aberrations, including *ADAM3A* (8p11.23) and *GSTT1* (22q11.23). In summary, our studies detected recurrent genetic alterations in NK/T-cell lymphoma, some of which are associated with adverse prognosis. Some candidate genes in these regions may be involved in the pathogenesis and disease progression.

## Introduction

Extranodal NK/T-cell lymphoma, nasal type (NK/TCL), is a highly aggressive disease. It occurs predominantly in adult male patients and most frequently involves the upper aerodigestive tract [1]. This disease shows ethnic predilection with a higher incidence in Asians, Mexicans, and South Americans. It accounts for 6.9 % of all non-Hodgkin lymphomas (NHLs) and 28.2 % of T- and NK-cell neoplasms in the Chinese population [2].

A few studies have described the cytogenetic alterations based on limited numbers of fresh or frozen tissues, but no specific changes have been achieved [3–7]. Recently, gene expression profiling studies and array comparative genomic hybridization (CGH) analyses revealed that NK/TCL showed frequent 2q gain and 6q16-25 loss [8–11]. Potential candidate tumor-suppressor genes in the 6q16-25 include *PRDM1*, *ATG5*, *AIM1*, and *HACE1* [8, 9]. However, molecular pathogenesis of NK/TCL remains unclear, likely due to the rarity of disease, the small biopsies, and the presence of abundant necrosis in the tumor. In this study, we performed array CGH analysis of 13 cases to define their genomic alteration patterns on formalin fixed and paraffin-embedded (FFPE) tissues. Recurrent genetic alterations were detected in NK/TCL cases. Our results may provide further insights into the genetic characteristics of NK/TCL.

## Materials and methods

Thirteen cases of NK/TCL were included in our study. This study was approved by Peking University Bioethics Committee and has therefore been performed in accordance with the ethical standards laid down in the 1964 Declaration of Helsinki and its later amendments. The diagnosis for each case was confirmed by a panel of expert hematopathologists according to 2008 WHO classification criteria, and these diagnoses were agreed upon by all expert reviewers in all 13 cases. The major antibodies used in this study included CD56, CD3ε, CD2, cytotoxic molecules (TIA-1, Granzyme B, Perforin), CD20 (Dako, Glostrup, Denmark), and TCRβF1 (Santa Cruz, CA, USA). In situ hybridization for Epstein-Barr virus encoded RNAs (EBER) (PanPath, Amsterdam, Holland) and PCR for T-cell receptor gamma gene (*TCRG*) rearrangement (BIOMED-2, InVivoscribe Technologies, Carladab, CA, USA) were performed in all cases. Reference samples were obtained from FFPE mucosal tissues with chronic inflammation.

Genomic DNA was extracted from 3–5 sections of 20-μm-thick tissue using DNeasy Blood and Tissue Kit (Qiagen, Valencia, CA, USA). Areas with necrosis and without tumor cells were dissected out from the specimen to make sure that the tumor cells accounted for >95 % of the remaining tissue. Genomic DNA concentration and purity were monitored using NanoDrop UV–VIS spectrophotometer (Thermo Scientific, USA), and the intactness of DNA was evaluated by electrophoresis on 2 % agarose gels. The extracted genomic DNA from all 13 cases met the following criteria: A260/A280 ratio within 1.8–2.0, A260/A230 ratio > 1.0, average DNA size > 500 bp, and fragmented DNA > 300 bp according to the published data [12].

Array CGH assays were performed using Agilent SurePrint G3 Human CGH Microarray Kit 2 × 400 K (Agilent G4448A, USA). Briefly, 2-μg genomic DNA and 2-μg reference DNA were labeled with ULS-Cy5 and ULS-Cy3 by ULS™ arrayCGH labeling kit (Agilent 5190-0419, USA), respectively. After purification, the same amount of labeled sample DNA and reference DNA was combined and then mixed with a provided hybridization solution and human Cot-1 DNA (Agilent 5190-3393, USA), followed by hybridization at 65 °C for 40 hrs, according to the manufacturer’s instructions. After two washings, the arrays were scanned on an Agilent Microarray Scanner. Feature Extraction Software (v9.5, Agilent, USA) was used for data extraction from raw microarray image files. Agilent CGH Analytics Software (v3.4) was used to visualize, detect, and analyze chromosomal patterns with microarray profiles. Chromosome X and Y were excluded because of sex mismatching.

Loss of genetic regions was confirmed using Sanger sequencing. Three alteration primers surrounding the identified variants were designed using Primer3 software (Table 1). Primers were tested for specificity of target region amplification using polymerase chain reaction (PCR) BLAST. PCR was carried out under standard conditions. Direct sequencing of the PCR product was performed on an ABI3130xl Genetic Analyzer (Applied Biosystems, Inc., Foster City, CA).

**Table 1:** Primer sequences and sequencing of the candidate genes residing in the frequently aberration regions

Candidate gene	CGH probe position	Primer sequence 5′–3′	Product position	Nucleotide substitution	Case no.
*ADAM3A*	102217–102276	Forward: TATGTTACCTGTTTTCACTCCCAGT	102137–102321	102240G > A	12
		Reverse: ACCATTCTACTTATGTGGGAGCA			
	108223–108282	Forward: TGTTTTAAACGTCCTACAACTGAAC	108111–108342	108225C > T	4, 5, 8, 9, 12, 13
		Reverse: TGATGCTATTGGTCATTCCTCCT			
				ins108114ATA	1, 2, 5, 6
*GSTT1*	6144–6203	Forward: ATTTCACTCTTGGCAAACATCAGGG	5993–6312	–	5, 6, 8, 12
		Reverse: GGAATGGCTTGCCTAAGACTTG			

*ADAM3A* disintegrin and metalloproteinase domain, *GSTT* glutathione S-transferase theta, *ins108114ATA* insertion of ATA into 108114 base pair

Patient survival was analyzed using a SPSS program (v17.0). The probability of overall survival (OS) was calculated using the Kaplan–Meier method with log-rank test for comparison. Significant difference was considered when the *p* value was <0.05.

## Results

### Clinicopathologic features

The clinicopathologic features of all 13 patients were summarized in Table 2. There were 10 males and 3 females with a median age of 41 years (28–60 years). Six patients presented with B symptom (fever, night sweat, or weight loss), 10 had a low-risk International Prognostic Index (IPI, 0 or 1), and 11 had a low Ann Arbor stage (stage I or II). The lymphoma involved nasal cavity in 8 cases. The other five cases occurred in the nasopharynx, oropharynx, duodenum, eyelid, and skin.

**Table 2:** Clinicopathological features of the study cohort

Case no.	Sex/age	Site of biopsy	Stage	IPI	Therapy	Response	Other sites involved	Follow-up	
								Status	Months
1	M/41	Oropharynx	III/IV	High risk	CHOP	NR	–	D	2
2	M/47	Nasopharynx	I/II	Low risk	RT	CR	–	A	66
3	M/31	Nasal cavity	I/II	Low risk	RT	NR	–	D	7
4	M/42	Nasal cavity	I/II	Low risk	RT	CR	–	D	30
5	M/33	Nasal cavity	I/II	Low risk	RT	PR	–	D	6
6	M/39	Nasal cavity	I/II	Low risk	RT	CR	–	D	53
7	F/28	Nasal cavity	I/II	Low risk	CHOP + RT	CR	–	A	60
8	F/36	Nasal cavity	I/II	Low risk	RT	NR	Orbit	D	6
9	M/41	Nasal cavity	I/II	Low risk	CHOP	NR	–	D	8
10	M/58	Duodenum	I/II	Low risk	CHOP	PR	–	D	24
11	M/48	Nasal cavity	III/IV	High risk	CHOP-like	PR	Testis	D	6
12	F/60	Skin	I/II	High risk	CHOP	NR	Nasal cavity	D	22
13	M/28	Eyelid	I/II	Low risk	CHOP-like	NR	–	D	2

*M* male, *F* female, *IPI* international prognostic index, *CHOP* cyclophosphamide, doxorubicin, vincristin, prednisone; *RT*, radiotherapy, *CR* complete regression, *PR* partial regression, *NR* no response, *A* alive, *D* dead

Low risk: IPI 0-1, High risk: IPI ≥ 2

All patients received CHOP or CHOP-like chemotherapy or/and radiotherapy. Four patients achieved complete regression (CR) with median survival of 8 months (range 2–66 months) (Fig. 2a), while 6 patients showed no responses to chemotherapy or radiotherapy. All the four CR patients received radiotherapy, and only one of them combined with CHOP chemotherapy.

**Fig. 1:**
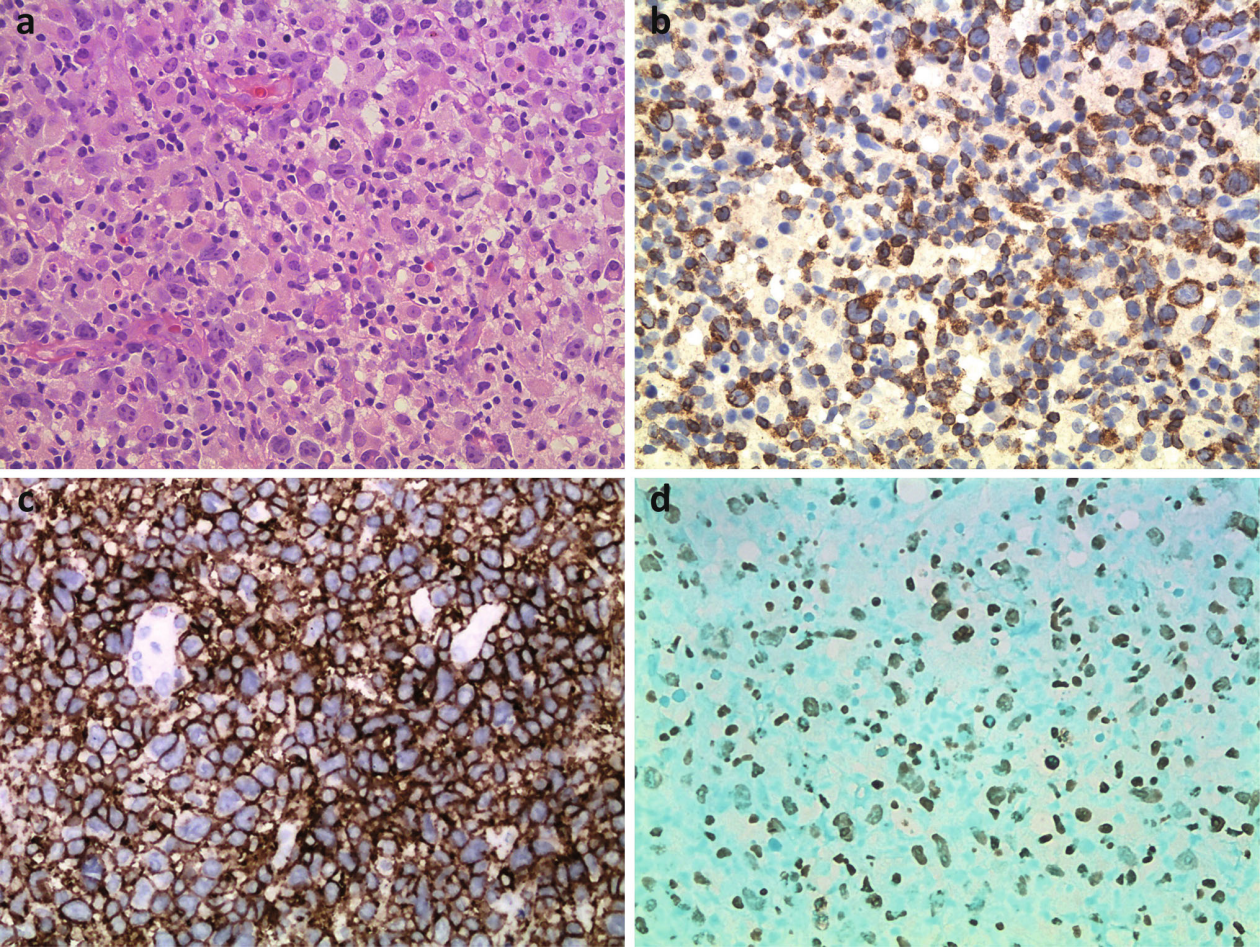
Pathological features of NK/TCL. Representative image of NK/TCL showing diffuse medium-sized to large and pleomorphic cell hyperplasia with a high mitotic rate (**a** H&E staining, ×400), positivity for CD3ε (**b** immunohistochemical staining, ×400), CD56 (**c** immunohistochemical staining, ×400), and EBER (**d** *in situ* hybridization, ×400)

Morphologically, the lymphomatous infiltrates were diffuse (Fig. 1a). An angiocentric and/or angiodestructive growth pattern was observed in 10 cases (10/13, 76.9 %). Coagulative necrosis was noted in 9 cases (9/13, 69.2 %), with a proportion of necrosis ranging from less than 5 % to 50 %. The tumor cells were positive for CD3ε (Fig. 1b) in 11 cases (11/13, 84.6 %) and positive for CD2 in 6 cases (6/13, 46.2 %). Twelve cases (12/13, 92.3 %) were positive for CD56 (Fig. 1c) and all cases positive for one or more cytotoxic molecules (TIA-1, Granzyme B or Perforin). EBV was detected in 11 of 13 cases by EBER in situ hybridization (Fig. 1d). No *TCRG* gene rearrangement was detected in all 13 cases. All cases were negative for CD20 and TCRβF1 immunostainings.

### Cytogenetic analysis

**Fig. 2:**
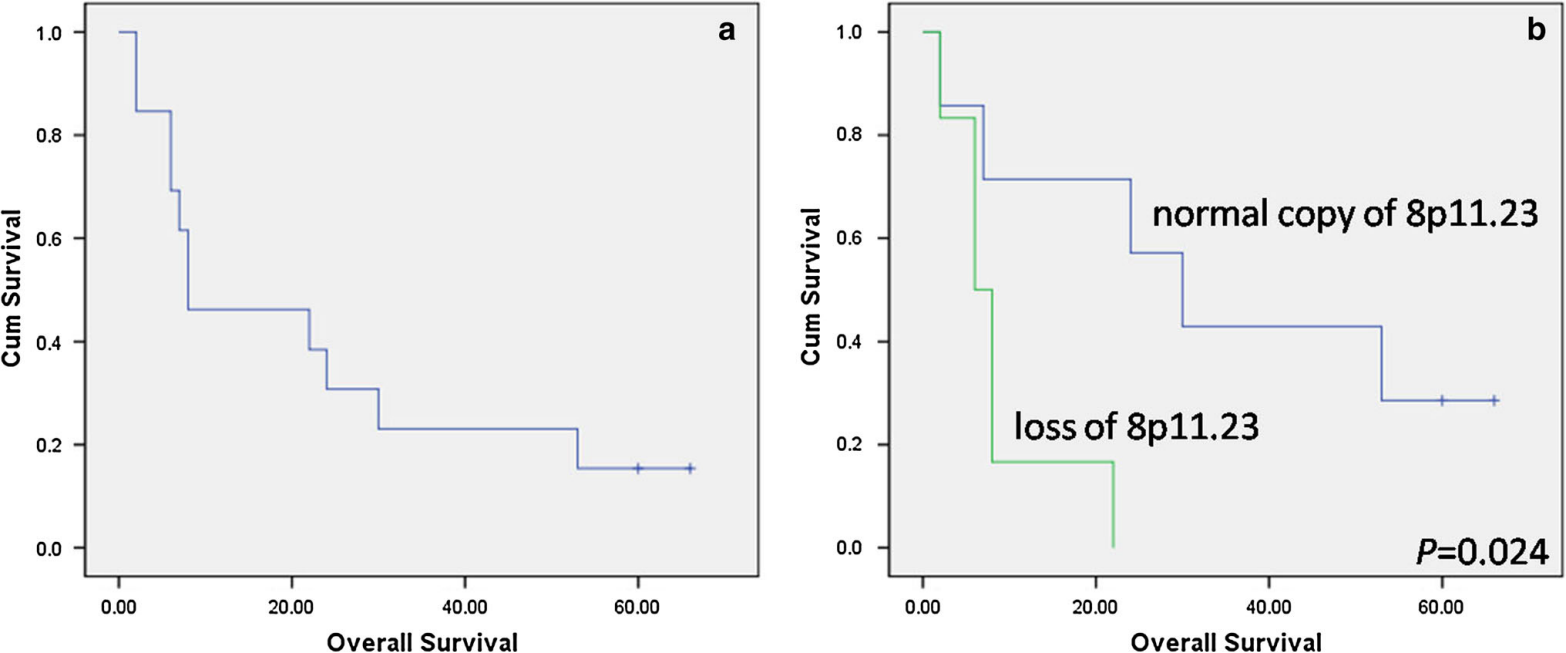
Overall survival of NK/TCL patients. **a** Overall survival of NK/TCL patients in the entire study cohort. **b** Overall survival of NK/TCL patients with or without loss of 8p11.23

By array CGH assays, we found 0–387 chromosomal aberrations, averaging 83, in the selected 13 cases of NK/TCL. The detailed information of recurrent genetic changes was listed in Table 3. There were a total of 177 recurrent chromosomal gains and 35 losses. Fourteen losses were detected in more than 30 % of the cases and 5 of them detected in over half of the cases, including losses of 15q24.2 (9/13, 69 %), 19q13.32 (9/13, 69 %), 5p13.2 (8/13, 62 %), 14q21.1 (8/13, 62 %), and 1q21.2 (7/13, 54 %) (Table 3). Eleven gains were detected in more than 30 % of the cases, including gains of 3q26.1 (6/13, 46 %), 7q34 (5/13, 38 %), and 8q24.3 (5/13, 38 %). Loss of 8p11.23 seemed to have a prognostic impact. Five out of six patients with this loss died within 8 months after the initial diagnosis with a median survival of 6 months versus 30 months for patients without this loss (*P* = 0.024) (Fig. 2b). Other chromosomal aberrations (gains or losses) did not correlate with patients’ prognosis.

**Table 3:** Recurrent chromosomal alterations in extranodal NK/T-cell lymphoma

	Cytogenetic band	Position (bp)	Candidate genes	No. of cases (case no.)
Loss	15q24.2	73419626–73419686	*COMMD4*	9 (3, 4, 5, 6, 8, 10, 11, 12, 13)
	19q13.32	53009579–53009634	–	9 (3, 4, 5, 6, 8, 9, 10, 12, 13)
	5p13.2	37523338–37523397	*WDR70*	8 (4, 5, 6, 8, 9, 10, 12, 13)
	14q21.1	37768843–37777108	–	8 (4, 5, 6, 8, 10, 11,12, 13)
	1q21.2	149245378–149245437	*FAM63A*	7 (3, 4, 5, 6, 10, 12, 13)
	3q21.1	124061602–124061661	*DIRC2*	6 (4, 5, 6, 8, 10, 12)
	8p11.23	39368509–39441901	*ADAM5P, ADAM3A*	6 (5, 8, 9, 11, 12, 13)
	13q21.1	52449301–52449360	–	6 (5, 6, 8, 10, 12, 13)
	15q11.2	19537035–20308073	*GOLGA8C, OR4M2, OR4N4, POTEB*…	5 (1, 4, 8, 10, 12)
	20p13	1516966–1532485	*SIRPB1*	5 (5, 6, 8, 10, 12)
	7q34	141699101–141711572	*TRY6*	5 (3, 6, 10, 11, 12)
	14q11.1-11.2	18798641–19446107	*OR4Q3, OR4M1, OR4N2, OR4K2…*	4 (4, 6, 10, 12)
	19q13.33	56828073–56840401	*SIGLEC14*	4 (8, 10, 11, 13)
	22q11.23	22682428–22712266	*GSTT1, GSTTP2, IGLL3, LRP5l*	4 (5, 6, 8, 12)
Gain	3q26.1	164101776–164101835	–	6 (3, 4, 6, 7, 9, 11)
	7q34	138381784–138416301	*ZC3HAV1*	5 (1, 4, 7, 10, 11)
	8q24.3	141602065–146144837	*PTK2, LY6* *K, LY6E, CYC1, HSF1…*	5 (1, 6, 7, 10, 11)
	1p31.1	71302816–72568068	*FUBP1, ZRANB2*	4 (1, 4, 7, 11)
	1q44	243072191–243106622	–	4 (1, 6, 7, 11)
	2q24.1	157968778–158039128	*CYTIP*	4 (7, 8, 10, 11)
	4q13.3	71740260–71751110	*ANKRD17, GRSF1, IGJ…*	4 (1, 7, 10, 11)
	4q27	122958722–122961366	*ANXA5, CCNA2*	4 (1, 7, 8, 11)
	6p21.32	32905664–33396254	*TAP2, TAP1, TAPBP*	4 (1, 7, 10, 11)
	6q14.1	76019791–76031723	*IBTK, PHIP, TMEM30A*	4 (1, 7, 10, 11)
	16p12.3	18751873–18772683	*SMG1*	4 (1, 7, 11, 13)

Among the 6 patients with 8p11.23 loss, 2 patients achieved partial regression (PR) and 4 patients showed no response (NR) to therapy. In the other 7 cases without 8p11.23 loss, there were 4 CR, 1 PR, and 2 NR.

**Fig. 3:**
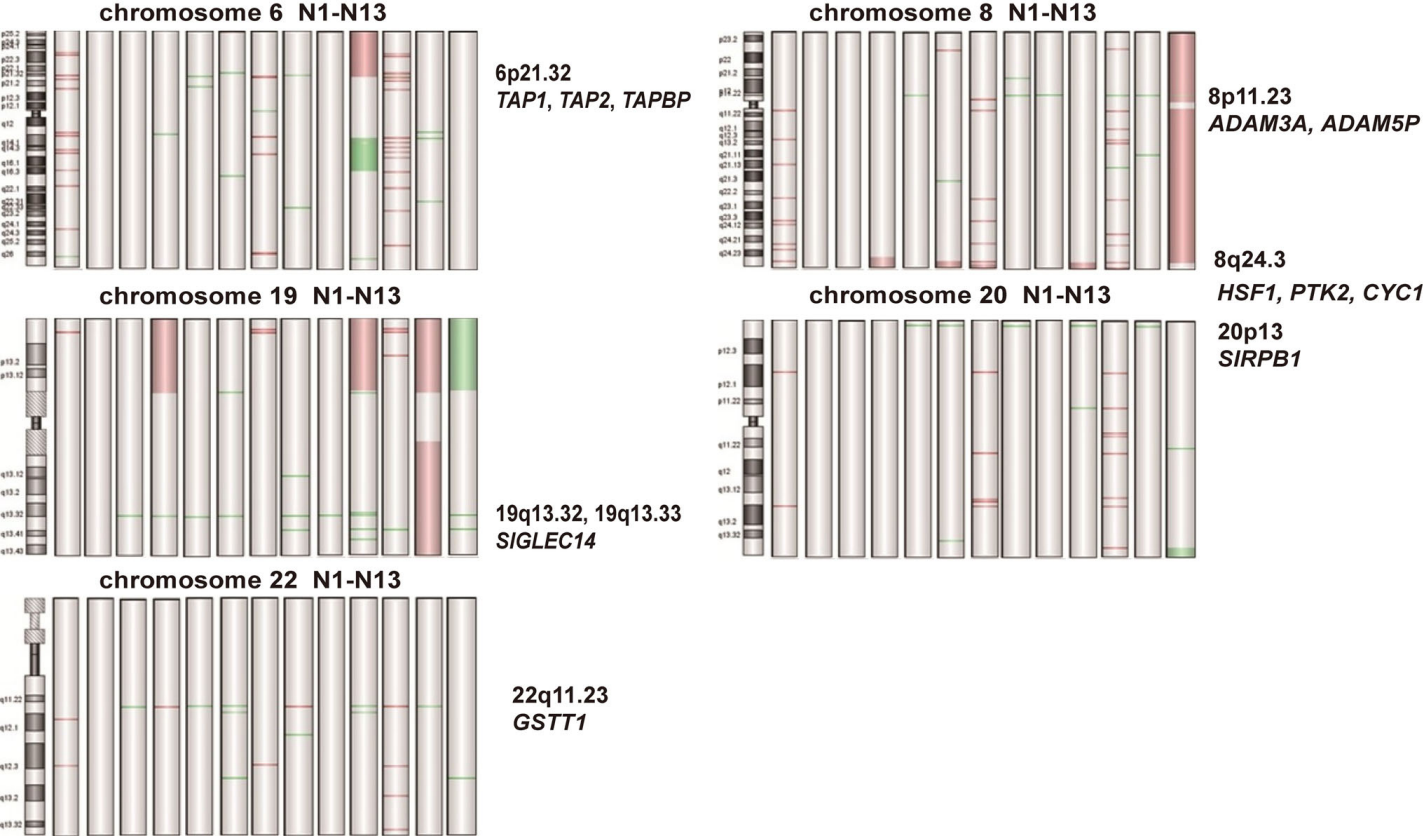
Ideograms of genomic alterations on chromosomes 6, 8, 19, 20, and 22. Genomic alterations are *underlined with* *green bars* for losses and *red bars* for amplifications marking the minimally involved chromosome regions

In the most recurrent regions (detected in >30 % of the cases, 11 gains and 14 losses), five regions (gains of 3q26.1 and 1q44, and losses of 19q13.32, 14q21.1, and 13q21.1) did not contain any known genes and protein-coding sequences (Table 3). The other 9 gain and 11 loss regions contained 194 and 24 known genes, respectively. Some of these genes were related to cell cycle, transcription or cell–cell and cell–matrix interaction, while most of them were functional unknown genes. Some candidate genes, including *ADAM3A*, *SIRPB1, SIGLEC14, GSTT1, TAP1, TAP2*, and *TAPBP*, may be related to the pathogenesis and progression of NK/TCL (Table 3). Their chromosome ideograms are shown in Fig. 3.

**Fig. 4:**
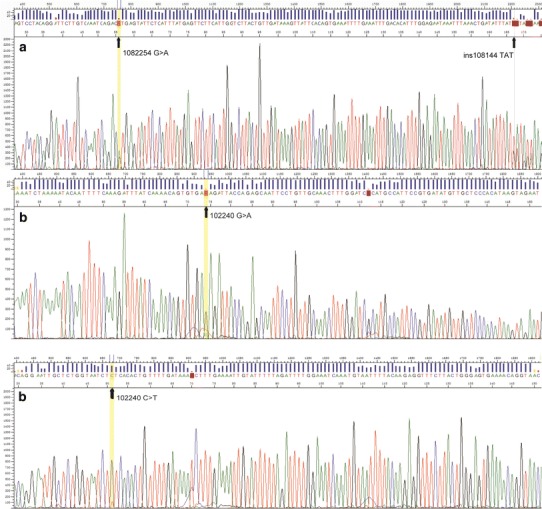
Sanger sequence of *ADAM3A*. **a** Sequence of the *ADAM3A* gene (position: 108223–108282 bp) of case 5 shows the 108225 G > A (C > T) mutation and insertion of TAT (ATA) into 108114 (ins108114) in reverse orientations. **b** Sequence of the *ADAM3A* gene (position: 102217–102276 bp) of case 12 shows the 102240 G > A (C > T) mutation in both forward (*upper of figure*) and reverse (*lower of figure*) orientations

The minimal deleted region across these six samples with 8p11.23 loss was the gene *ADAM3A and ADAM5P*. The 22q11.23 region contained genes including *GSTT1*, *GSTTP2*, *IGLL3*, and *LRP5l*. The *ADAM3A* and *GSTT1* genes were then further analyzed using Sanger sequencing of their CGH probe regions. A breakdown of the type and distribution of the point mutations identified is given in Table 1. One single-nucleotide substitution 108225 C > T in the *ADAM3A* gene was the most common variation that was detected in 5 of these 6 cases (Cases 5, 8, 9, 12, and 13, Fig. 4a). In addition, insertion of ATA into 108114 was also found in one of these cases (Case 5), while 102240 G > A was found only in Case 12 (Fig. 4b). There was no amplification of product in the 4 cases with 22q11.23 loss (Table 1), while a normal *GSTT1* sequence was found in the other cases.

## Discussion

NK/TCL is highly prevalent in Asians and is the most common type of NK/T-cell neoplasms in China [2]. It typically occurs in adult males and most commonly involves the nasal cavity. Pathologically, it is characterized by vascular damage, prominent necrosis, cytotoxic immunophenotype and association with Epstein-Barr virus. NK/TCL is an aggressive lymphoma with a poor prognosis. In recent years, a few studies have reported some chromosomal aberrations and identified some candidate tumor-suppressor genes in NK/TCL [8–11], but no definitive conclusion has been reached. In this study, array CGH assays were used to detect the cytogenetic characteristics of NK/TCL in order to investigate the common chromosomal aberrations and to find the candidate genes that might be related to the pathogenesis and progression of NK/TCL.

Twenty-five chromosomal abnormalities including 11 gains and 14 losses were detected in more than 30 % of the cases in this study (Table 3). Gains of 1q44, 6p21.31, and 7q34 and loss of 13q21.1 had been described previously in NK/TCL [3, 9], but their significance and the candidate genes located in these regions were still unclear. Four cases showed gain of 6p21.31, a region containing *TAP1*, *TAP2*, and/or *TAPBP* genes. *TAP* involves the peptides transportation from cytoplasm into endoplasmic reticulum (ER) lumen and then loading onto newly synthesized MHC class I molecules [13]. Ressing et al. demonstrated that BNLF2a (EBV-encoded protein) prevented the import of peptides into the ER by *TAP* by blocking the binding of peptides with ATP to the transporter complex [14]. Losses of 8p11.23 and 20p13 were detected in 6 and 5 cases, respectively. Although losses of these two regions have not been reported in NK/TCL, Flossbach et al. [15] found these two aberrations in extranodal marginal zone lymphoma of mucosa-associated lymphoid tissue (MALT lymphoma) using single-nucleotide polymorphism. Gain of 8p11.23 was observed in small cell variant of MALT lymphoma, whereas composite variant had both gain and loss of 8p11.23 and large cell variant only showed loss of this sequence, reflecting the association of this aberration with lymphoma progression. They also found that gain of 20p13 was related to progression of MALT lymphoma as well [15]. Gain of 20p13 was found in follicular lymphoma by Boonstra et al. [16]; however, its function was unclear. In this study, only losses of 8p11.23 and 20p13 were detected, without gains of these regions. Interestingly, patients with loss of chromosome 8p11.23 showed a worse prognosis than patients without this loss, with a much shorter OS (*P* = 0.024), and 5 out of 6 patients died within 8 months after initial diagnosis. The chromosomal 8p11.23 region contains *ADAM3A* gene, a member of disintegrin and metalloproteinase (*ADAM*) family, which is widely expressed and has many potential functions related to cell–cell and cell–matrix interactions [17]. Several members of this family have been implicated in cancer [18]. Using Sanger sequencing, we attempted to validate CGH data with two specific *ADAM3A* gene primers. A single-nucleotide mutation at the 108225 base pair (bp) was detected in 5 of the 6 cases with 8p11.23 loss, and one of these 5 cases (Case 12) also showed another single-nucleotide mutation at the 102240 bp (Fig. 4). As a general rule, array CGH does not detect changes in DNA smaller than 80 kb, but point mutation may influence hybridization efficiency or result in failure of hybridization in the case of high hybridization temperature (65 °C in our study). Mutation of the *ADAM3A* gene may decrease protein expression so as to change the intercellular adhesion, which may potentially affect the tumor microenvironment. The definitive role of *ADAM3A* gene in NK/TCL remains to be further studied.

Aberrations of 22q11.23 were detected in 7 cases, including loss in 4 cases and gain in 3 cases. This region contains *GSTT1* gene, belonging to Glutathione S-transferase (*GST*) family, which consists of several phase II detoxification enzymes and plays important roles in detoxification of a wide range of substrates, including mutagens, carcinogens, and chemotherapeutic agents, such as alkylating agents, platinum compounds, and anthracyclines. Besides its important roles in protecting cells from environment, genotoxic and oxidative stress, the detoxification capability is also associated with drug resistance [19]. The loss of *GSTT1* was reported to be associated with an increased risk of Hodgkin lymphoma and some types of non-Hodgkin lymphoma. However, it is intriguing that loss of *GSTT1* is associated with a better prognosis in patients treated with substrates for *GSTT1* [20]. A possible explanation is that the loss of *GSTT1* decreases the detoxification capability so as to decrease the pharmacokinetic and pharmacodynamic profile of chemotherapeutic agents, leading to a better response to treatment. In our study, the aberrations of *GSTT1* gene in NK/TCL were various, which presented as loss and gain. We attempted to detect the *GSTT1* gene in cases with 22q11.23 loss. There was no amplification of product in any of the 4 cases with 22q11.23 loss (Table 1), while a normal *GSTT1* sequence was found in the other cases. This *GSTT1*’s contribution to NK/TCL remains unclear and needs more studies.

In summary, the present array CGH results suggested the involvement of a complex and multiple genetic changes in the development of NK/TCL. A further study to identify the potential candidate genes was required to better understand the pathogenesis of NK/TCL.
